# Progress of the Cholera

**Published:** 1849-06

**Authors:** 


*Progress of the Cholera.*—Epidemic Cholera has made its appearance at several different points since our last number was issued. We have not means of obtaining much information respecting its progress, in addition to that communicated by the Newspaper press, and the latter, of course, is equally available for all our readers. We hope that in every place in which it prevails, some one, or more, will take pains to collect and record important facts relating to the disease, in order to supply data for an accurate history of the Epidemic. Five cases of the disease have occured in this city up to the present date, (June 7th,) two of which only were indigenous. The remainder were attacked on board vessels, and were from places where the Epidemic was prevalent (Chicago and Cincinnati.) Only one of these cases have proved fatal. The two cases developed here, had no connection with the cases imported, there being no means of communication between them.

